# The structure of the GemC1 coiled coil and its interaction with the Geminin family of coiled-coil proteins

**DOI:** 10.1107/S1399004715016892

**Published:** 2015-10-31

**Authors:** Christophe Caillat, Alexander Fish, Dafni-Eleftheria Pefani, Stavros Taraviras, Zoi Lygerou, Anastassis Perrakis

**Affiliations:** a Department of Biochemistry, The Netherlands Cancer Institute, 1066 CX Amsterdam, The Netherlands; b Laboratory of Biology, School of Medicine, University of Patras, 26505 Rio, Patras, Greece; c Laboratory of Physiology, School of Medicine, University of Patras, 26505 Rio, Patras, Greece

**Keywords:** Geminin, GemC1, Idas, McIdas, multicilin, coiled coil, DNA-replication license, protein stability

## Abstract

GemC1, together with Idas and Geminin, an important regulator of DNA-replication licensing and differentiation decisions, constitute a superfamily sharing a homologous central coiled-coil domain. To better understand this family of proteins, the crystal structure of a GemC1 coiled-coil domain variant engineered for better solubility was determined to 2.2 Å resolution. GemC1 shows a less typical coiled coil compared with the Geminin homodimer and the Geminin–Idas heterodimer structures. It is also shown that both *in vitro* and in cells GemC1 interacts with Geminin through its coiled-coil domain, forming a heterodimer that is more stable that the GemC1 homodimer. Comparative analysis of the thermal stability of all of the possible superfamily complexes, using circular dichroism to follow the unfolding of the entire helix of the coiled coil, or intrinsic tryptophan fluorescence of a unique conserved N-terminal tryptophan, shows that the unfolding of the coiled coil is likely to take place from the C-terminus towards the N-terminus. It is also shown that homodimers show a single-state unfolding, while heterodimers show a two-state unfolding, suggesting that the dimer first falls apart and the helices then unfold according to the stability of each protein. The findings argue that Geminin-family members form homodimers and heterodimers between them, and this ability is likely to be important for modulating their function in cycling and differentiating cells.

## Abstract 2

The GemC1 coiled-coil structure has subtle differences compared with its homologues Geminin and Idas. Co-expression experiments in cells and biophysical stability analysis of the Geminin-family coiled coils suggest that the GemC1 coiled coil alone is unstable.

## 1. Introduction   

Geminin coiled-coil domain-containing protein 1, GemC1, is a member of the Geminin superfamily. The three members of this family, Geminin, Idas and GemC1, all share a conserved coiled-coil domain.

Geminin was the first to be identified, as an inhibitor of DNA replication (McGarry & Kirschner, 1998 ▸; reviewed in Caillat & Perrakis, 2012 ▸). The binding of Geminin to Cdt1 inhibits the loading of the mini-chromosome maintenance complex (MCM) onto chromatin and pre-replication complex (preRC) formation (Tada *et al.*, 2001 ▸; Wohlschlegel *et al.*, 2000 ▸; reviewed in Lygerou & Nurse, 2000 ▸; Symeonidou *et al.*, 2013 ▸). Besides its role in proliferation, Geminin also has a role in cell differentiation (Seo & Kroll, 2006 ▸; Champeris Tsaniras *et al.*, 2014 ▸). The coiled coil of Geminin resides in the middle of the protein and assembles in a head-to-head coiled-coil homodimer that binds one molecule of Cdt1 (De Marco *et al.*, 2009 ▸; Lee *et al.*, 2004 ▸; Saxena *et al.*, 2004 ▸).

Idas (also referred to as multicilin and McIdas) was identified as a protein that interacts with Geminin, exhibits high levels of expression in the mouse forebrain and regulates DNA replication and centrosome numbers (Pefani *et al.*, 2011 ▸). Idas has also been identified as a key regulator of multiciliate cell differentiation that drives centriole biogenesis (Ma *et al.*, 2014 ▸; Stubbs *et al.*, 2012 ▸). Idas preferentially interacts with Geminin than with itself, forming a tight heterodimer between the two coiled-coil domains (Caillat *et al.*, 2013 ▸).

GemC1 has been identified as a Geminin homologue, and is also implicated in DNA replication but at a later stage than Geminin. GemC1 has been shown to mediate TopBP1- and Cdk2-dependent recruitment of Cdc45 onto replication origins, enabling pre-initiation complex formation and initiation of DNA replication (Balestrini *et al.*, 2010 ▸).

The mechanism by which Geminin is able to coordinate both cell proliferation and cell differentiation is not fully understood (Caillat & Perrakis, 2012 ▸; Champeris Tsaniras *et al.*, 2014 ▸). Having previously shown that Idas can preferentially interact with Geminin through its coiled-coil domain and that this interaction is important for Idas function (Caillat *et al.*, 2013 ▸; Pefani *et al.*, 2011 ▸), we sought to examine the structure of GemC1 and how this might explain its function and the relationships within the Geminin family.

## 2. Materials and methods   

### 2.1. Cloning, expression and purification   

A synthetic gene (GenScript) harbouring a codon-optimized DNA sequence (according to the manufacturer’s protocols) was used for all human GemC1 (UniProt ID A6NCL1) constructs. The constructs for GemC1, tGemC1 (64–146), dGemC1 (29–240), GemC1_C-ter (241–334) and full-length GemC1 (1–334), were cloned into the pETNKI-His-3CLIC-kan vector (Luna-Vargas *et al.*, 2011 ▸) for expression with a cleavable His tag. The constructs for Geminin, full-length Geminin, dGeminin (29–209) and tGeminin (82–160), and the Idas construct used for the purification of tIdas–tGeminin and tIdas–tIdas dimers have been described previously (Caillat *et al.*, 2013 ▸; De Marco *et al.*, 2009 ▸). To express the tGemC1–tGeminin heterodimer, we used the tGemC1 construct described above together with a tGeminin construct that we have described previously (Caillat *et al.*, 2013 ▸) and inserted it into the pET-22b (Novagen) vector for expression without a tag. As these two plasmids are resistant to kanamycin and ampicillin, respectively, they allow efficient co-expression experiments. The two mutations in GemC1, L123E and L130E (tGemC1^L123,130E^), were generated using the QuikChange site-directed mutagenesis kit (Stratagene). All complexes were purified by IMAC and size-exclusion chromatography in a buffer consisting of 50 m*M* HEPES–NaOH pH 7.5, 150 m*M* NaCl, 0.5 m*M* TCEP; detailed protocols are available in Caillat *et al.* (2013 ▸) and De Marco *et al.* (2009 ▸). We note that the heterodimers are purified with a tag on either GemC1 or Idas; as untagged Geminin is the more abundantly expressed protein in our experiments, purification of the less abundant protein practically ensures purification of the heterodimer. All proteins were further purified by size-exclusion chromatography and the final product was examined by Coomassie Brilliant Blue-stained polyacrylamide gel electrophoresis to confirm that an approximately stoichiometric amount of complex was the final purification product.

### 2.2. Multi-angle laser light scattering   

Multi-angle laser light scattering (MALLS) experiments were performed in a Superdex 75 HR 10/30 column attached to an ÄKTA FPLC and coupled to a miniDAWN light-scattering detector (Wyatt Technology) and a Dn-1000 differential refractive-index detector (WGE Dr Bures). 100 µl of purified tGemC1 dimer at a concentration of ∼2.0 mg ml^−1^ were injected onto the column. Data analysis was carried out with *ASTRA* using a *d*n/*d*c value of 0.185. Size-exclusion chromatography runs for tGemC1–tGeminin were performed in a Superdex 75 HR 10/30 column attached to an ÄKTApurifier.

### 2.3. Mammalian cell culture, transfection and immunoprecipitation   

HA-tagged GemC1, Geminin-GFP, Geminin(1–72)-GFP, Idas-GFP and Cdt1-GFP were cloned in pcDNA3.1 for expression in mammalian cells. U2OS cells were cultured in DMEM (Invitrogen) with 10% foetal bovine serum (Invitrogen). Cells were transfected with the TurboFect transfection reagent (Fermentas) according to the manufacturer’s instructions. U2OS cells were transfected with GEMC1-HA and other constructs as indicated and were collected 24 h post-transfection. Immunoprecipitation of GEMC1-HA was performed using an anti-HA antibody (12CA5, Santa Cruz) as described in Pefani *et al.* (2011 ▸). Immunoprecipitates and total cell extracts corresponding to 10% of immunoprecipitates were analysed by Western blotting using anti-HA (Molecular Probes), anti-GFP and anti-Geminin (Xouri *et al.*, 2004 ▸; Iliou *et al.*, 2013 ▸) antibodies.

### 2.4. *T*_m_ determination based on tryptophan fluorescence (OPTIM 1000)   

Thermal unfolding and aggregation curves were measured in 25 m*M* HEPES–NaOH pH 7.5, 150 m*M* NaCl, 0.5 m*M* tris(2-carboxyethyl)phosphine at a concentration of 1 mg ml^−1^ using an OPTIM 1000 from Avacta.

The barycentric mean fluorescence was calculated according to
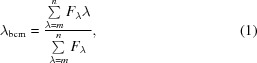
where λ_bcm_ is the barycentric mean, λ is the wavelength, *F*_λ_ is the fluorescence intensity at wavelength λ, *m* = 300 nm and *n* = 450 nm.

The static light-scattering signal was also recorded from the samples to detect the presence of aggregates.

### 2.5. Analysis of the stability of the coiled coil by circular dichroism (CD)   

Far-UV CD experiments were performed on a J-810 spectropolarimeter (Jasco) with a Peltier thermocontrol element (Jasco). CD data were recorded at a fixed wavelength of 220 nm with a linear temperature gradient from 10 to 90°C. All samples were adjusted to a concentration of ∼0.3 mg ml^−1^. No visual precipitation was observed after completion of the experiment. Data analysis was performed using the formulae described in Greenfield (2006 ▸) as implemented in *GraphPad Prism* by the authors.

### 2.6. Crystallization   

Screening was performed using previously described procedures (Newman *et al.*, 2005 ▸) in 96-well sitting-drop vapour-diffusion plates (MRC 2-Well Crystallization Plate manufactured by Swissci). Following optimization, crystals used for diffraction studies were grown at 4°C, mixing 200 nl 10 mg ml^−1^ tGemC1^L123,130E^ with 200 nl 0.1 *M* HEPES buffer pH 7.5, 7% ethanol, 10% 2-methyl-2,4-pentanediol (MPD), 0.01 *M* ethylenediaminetetraacetic acid disodium salt dihydrate. Crystals were soaked in the reservoir solution supplemented with MPD to a final concentration of 32%(*w*/*v*) and were vitrified by plunging into liquid nitrogen.

### 2.7. Data collection, structure solution and refinement   

Diffraction data were collected on beamline ID23-2 at the ESRF at a wavelength of 0.8726 Å. Intensity integration and scaling was performed using the *XDS* package (Kabsch, 2010 ▸). The structure was solved by molecular replacement with *Phaser* (McCoy, 2007 ▸) using a polyalanine model of dimeric Geminin (PDB entry 2wvr; De Marco *et al.*, 2009 ▸) as the search model. One homodimer of tGemC1^L123,130E^ was present in each asymmetric unit of the *P*2_1_2_1_2_1_ unit cell. The model was rebuilt in the map resulting from the molecular-replacement solution using *ARP*/*wARP* (Langer *et al.*, 2008 ▸) and manually adjusted in *Coot* (Emsley *et al.*, 2010 ▸). Refinement was performed using *phenix.refine* (Afonine *et al.*, 2012 ▸) and in later stages using the *PDB_REDO* web server (Joosten *et al.*, 2014 ▸) incorporating *REFMAC* (Murshudov *et al.*, 2011 ▸). Statistics of data reduction and structure refinement are presented in Table 1 ▸.

## 3. Results and discussion   

### 3.1. The structure of the GemC1 coiled coil   

**Figure 1:**
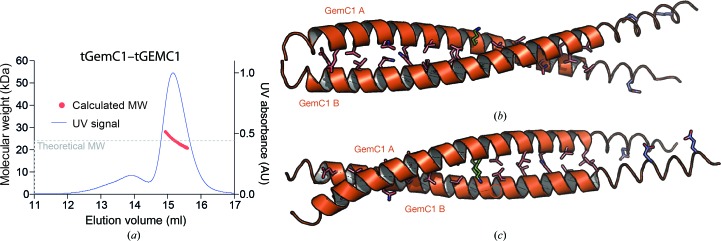
The structure of the GemC1 dimer. (*a*) Size-exclusion chromatography and multi-angle laser light-scattering measurements of the tGemC1 homodimer. The mean molecular weight per volume unit (red line) and the normalized UV_280 nm_ elution profile (blue line) are shown. The theoretical molecular weight for the dimer is represented as a grey dashed horizontal line. Graphs are representative of at least two experiments. (*b*, *c*) The structure is shown as an orange cartoon, with a thick tape model for the region spanning the formal coiled coil, and regions that do not conform with the coiled-coil formalism shown as a thin ribbon. (*b*) and (*c*) are rotated 90° along the horizontal viewing axis with respect to each other. Residues in the *a* and *d* positions are shown as sticks (oxygen, red; nitrogen, blue). Lys97, which does not form a ‘knobs-into-holes’ interaction, is depicted as sticks with C atoms in green. The positions of the L123E and L130E solubility-enhancing mutants towards the C-terminus are also shown as sticks with C atoms in light blue.

Expression trials of full-length GemC1 (1–334) and an extended construct encompassing a ‘long’ predicted coiled-coil domain (29–208; dGemC1) resulted in insoluble protein. Expression of a construct slightly longer than the predicted coiled-coil domain (64–146; tGemC1) resulted in protein that was soluble at concentrations below 1 µ*M*. Several tags (GST, SUMO and Trigger Factor) did not improve the solubility. Examining the helical wheel prediction diagram of the GemC1 coiled-coil homodimer, we observed that some hydrophobic residues are not in the core interface (register positions *a* and *d*), but are instead exposed to the solvent. In particular, at the C-terminal end of the coiled coil, residues Leu119, Val120, Leu123, Ala127, Leu130 and Leu131 constitute a hydrophobic patch. We hypothesized that this hydrophobic patch could lead to aggregation of the GemC1 protein. We thus decided to mutate residues Leu123 and Leu130 to glutamates to increase the solubility. It should be noted that some predictions place these residues in the *d* position of the coiled coil; however, these predictions assume a coiled-coil irregularity in the 113–114 region, something that is unlikely based on the structures of the homologous coiled coils of Geminin and Idas. Our mutations yielded the construct tGemC1^L123,130E^, which allowed the expression of highly soluble protein (>2 m*M*). The protein behaved as a dimer in a size-exclusion chromatography coupled to multi-angle laser light scattering (MALLS) experiment (Fig. 1 ▸ *a*).

tGemC1^L123,130E^ (from here on we will refer to this construct as tGemC1 for simplicity) was crystallized and the structure was determined to 2.2 Å resolution and refined to an *R*_free_ of 24.9% with excellent geometry (Table 1 ▸). The structure showed a typical dimeric parallel coiled-coil homodimer (Figs. 1 ▸ *b* and 1 ▸ *c*), with two α-helices that pack together in a left-handed superhelix. Both chains have about 20 disordered residues in the C-terminus and five disordered residues in the N-terminus, as only residues 69–132 and 69–129 are well resolved in the electron density in each of the two chains. Residues 71–129 and 70–124 are in α-helical conformation in each chain. The two mutated leucine residues are indeed pointing to the solvent, as expected from our sequence analysis and in contrast to the other predictions discussed above. Although we cannot formally exclude that our mutations changed the coiled coil, this is very unlikely as we observe regular helices and the coiled coil stops in approximately the same place as in the homologous structures of the Geminin (PDB entry 1uii; Thépaut *et al.*, 2004 ▸) and Geminin–Idas (PDB entry 4bry; Caillat *et al.*, 2013 ▸) coiled coils. Based on our structural data, we conclude that the change of the hydrophobic solvent-exposed Leu123 and Leu130 to hydrophilic glutamate residues improved solubility without affecting the global structure.

Analysis of the structure using the *SOCKET* software (Walshaw & Woolfson, 2001 ▸) shows that the coiled-coil region extends from residues 73 to 115 and spans six heptads (technically speaking, one residue of a seventh heptad is present). In position *d*_4_, Lys97 does not form a ‘knobs-into-holes’ interaction, forming a minor but characteristic irregularity in the series of interactions in the length of the coiled coil (Fig. 1 ▸ *c*).

**Figure 2:**
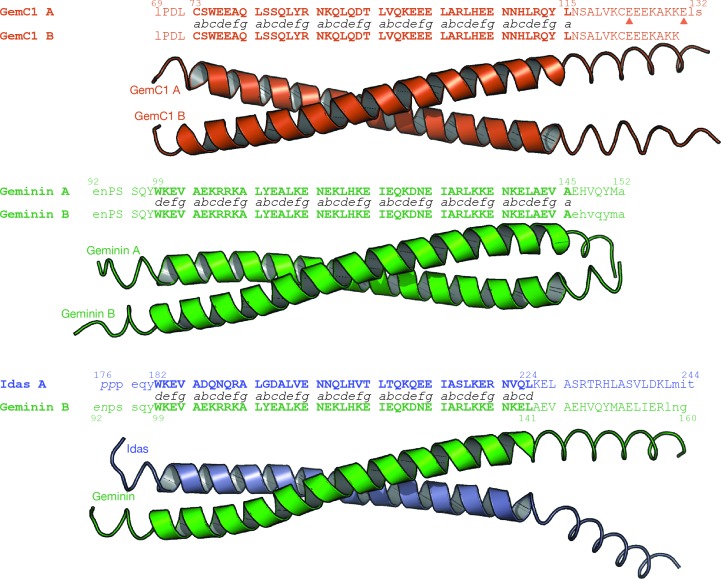
Comparison of the structures of the GemC1 dimer (orange), the Geminin dimer (green) and the Idas–Geminin heterodimer (blue and green). The three dimeric structures have been aligned together using the *RAPIDO* server. The optimal alignment we present here uses the coiled-coil region of the monomer in chain *A*. The cartoon representation is as in Fig. 1 ▸. The sequence alignment of the three dimers is also shown, with residues in the coiled-coil region as bold upper-case letters, residues in an α-helical conformation as upper-case letters and residues outside the helix as lower-case letters.

The structure of the Geminin coiled-coil homodimer as well as the structure of the Geminin–Idas coiled-coil heterodimer have previously been determined (Thépaut *et al.*, 2004 ▸; Caillat *et al.*, 2013 ▸). Comparing these structures with that of the tGemC1 homodimer (Fig. 2 ▸) shows several interesting features. Firstly, all three structures are composed of coiled coils of similar length, with Geminin having a more extended coiled coil (six full heptads with a four-residue N-terminal extension and a one-residue C-terminal extension) and Idas–Geminin a less extended coiled coil (five core heptads with two N- and C-terminal flanking regions of four residues each); GemC1 is intermediate in length. Analysis of the coiled-coil parameters by the program *CCCP* (Grigoryan & Degrado, 2011 ▸) shows that the GemC1 coiled coil has an ω_0_ angle of −4.1° per residue, suggesting a relatively tight left-handed superhelix compared with the Geminin homodimer (ω_0_ = −3.9° per residue) and Idas–Geminin (ω_0_ = −3.7° per residue). The superhelical radius (the distance from the superhelix axis to the helical axis of the chains) is longer in GemC1 at 5.1 Å compared with 4.7 Å for both Geminin and Idas–Geminin. The surface buried at the interface of GemC1 (1370 Å^2^) is slightly less than for Idas–Geminin (1463 Å^2^) and Geminin–Geminin (1572 Å^2^).

**Figure 3:**
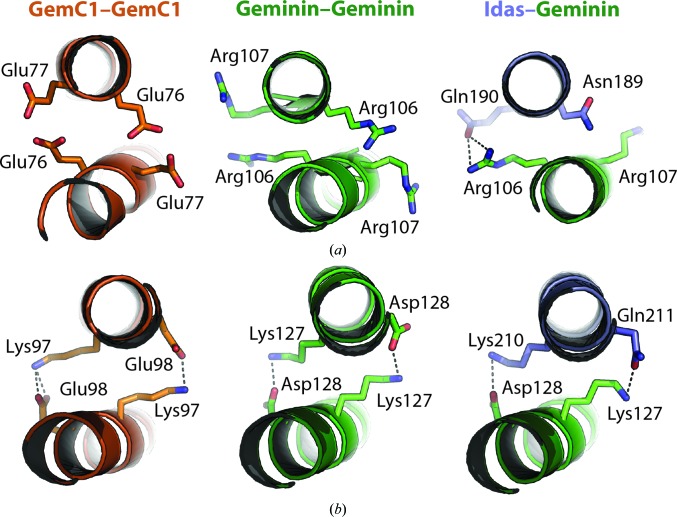
Different unusual residues in the *d*_1_ (*a*) and *d*_4_ (*b*) positions of GemC1, Geminin and Idas–Geminin. Representation and colouring is as in Figs. 1 ▸ and 2 ▸.

Similarly to both Geminin and Geminin–Idas, GemC1 has several nonhydrophobic residues in the *a* and *d* register positions: *d*_1_, *d*_2_, *a*_1_, *d*_4_ and *a*_6_. In addition, GemC1 has a highly unusual cysteine residue at position *a*_1_ (an alanine in both Geminin and Idas). The residue in position *d*_1_ is the negatively charged Glu76 in GemC1 and is followed by Glu77; this is sharply opposed to the positively charged pair of Arg106 and Arg107 residues in Geminin and the Asn189 and Gln190 polar pair in Idas (Fig. 3 ▸ *a*). The Glu76 in GemC1 creates an electrostatic repulsion with Glu77 from the second monomer in GemC1, resulting in the two helices of the coil being further apart than in the other structures. In position *d*_2_, GemC1 has a Gln in place of an Ala in the other two structures. This Gln83 is involved in a nonsymmetric network of side-chain inter­actions that also involves the well conserved Asn87 in position *a*_1_. The Lys97 residue in position *d*_4_, which is fully conserved in Geminin and Idas, interacts with Glu98 in position *e*_4_ of the opposing chain; in the Geminin and Idas structures this is Asp128 (Fig. 3 ▸ *b*). Apparently, maintaining the hydrogen-bonding interaction with the longer Glu98 places Lys97 in GemC1 in a more extended conformation that is incompatible with the definition of the ‘knobs-into-holes’ geometry for coiled coils (see above), but still maintains hydrogen-bonding interactions between the monomers, suggesting that this residue is not crucial for coiled-coil formation and that the lack of the ‘knobs-into-holes’ structure is rather an anomaly and not a defining feature of the GemC1 coiled coil. Finally, the Asn108 in position *a*_6_ is conserved in the family and is involved in a stabilizing hydrogen bond between the two chains.

### 3.2. GemC1 and Geminin interact through the coiled-coil domain   

**Figure 4:**
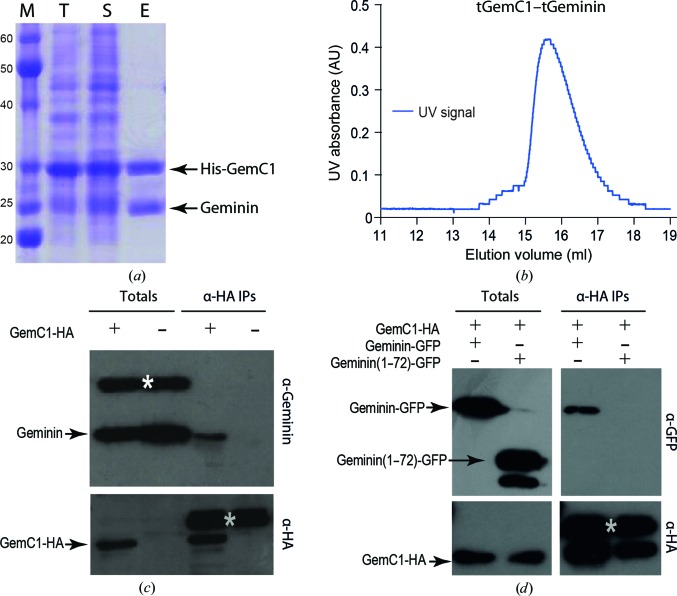
GemC1 heterodimerizes with Geminin. (*a*) Co-expression of His-tagged dGemC1 (29–240) and untagged dGeminin (29–209) in *E. coli*. GemC1 is the best overexpressed protein both in the total cell lysate (lane T) and the supernatant (lane S). However, purification by Ni^2+^ affinity results only in an approximately stoichiometric GemC1–Geminin complex (lane E). Lane M contains molecular-weight markers (labelled in kDa). (*b*) Size-exclusion chromatography of the tGemC1–tGeminin heterodimer showing the normalized UV_280 nm_ elution profile (blue line). (*c*) HA-tagged GemC1 was overexpressed in U2OS cells and was able to co-precipitate endogenous Geminin, suggesting that the two proteins also interact in human cells; in the lower panel the grey star marks the large chain of the IgGs present in the anti-HA immunoprecipitates; the white star in the upper panel marks a band cross-reacting with the Geminin antibody. (*d*) HA-tagged GemC1 was overexpressed in U2OS cells in the presence of either GFP-tagged Geminin or a construct of Geminin lacking the coiled-coil domain, Geminin(1–72), indicating that the Geminin coiled coil is necessary for interaction with GemC1 in U2OS cells; the grey star marks the large chain of the IgGs present in the anti-HA; the unlabelled band below Geminin(1–72)-GFP is most likely to be a degradation product.

We have previously shown that Idas prefers to interact with Geminin and form a heterodimer than to homodimerize (Caillat *et al.*, 2013 ▸; Pefani *et al.*, 2011 ▸). To determine whether the same holds true for GemC1, we first co-expressed His-tagged GemC1 and Geminin and were able to purify a stoichiometric complex between the two proteins (Fig. 4 ▸ *a*). This is also notable because GemC1 alone was never soluble in our expression trials. In addition, the coiled coil of Geminin (tGeminin) was sufficient to solubilize the coiled-coil domain of GemC1 (tGemC1) and of the longer dGemC1, but was not sufficient to solubilize full-length GemC1. These results indicate that GemC1 and Geminin interact through their coiled-coil domains but are likely to have more extended inter­actions, as full-length GemC1 needs full-length Geminin to stabilize. Notably, even when GemC1 is more abundant than Geminin in the cell lysates purification through the His tag attached to GemC1 results in an approximately stoichiometric 1:1 complex between GemC1 and Geminin (Fig. 4 ▸ *a*), suggesting that at least under these specific conditions the GemC1–Geminin complex is preferred. Finally, expression and purification of the tGemC1–tGeminin complex (by IMAC on the His tag on GemC1 alone) resulted in a complex that subsequently ran as a single peak on a size-exclusion chromatography column (Fig. 4 ▸ *b*), with a retention volume directly comparable to that of the tGemC1–tGemC1 homodimer (Fig. 1 ▸ *a*), suggesting that tGemC1 and tGeminin fold as a stable stoichiometric heterodimer.

To test whether GemC1 also interacts with Geminin in human cells, we transfected U2OS cells with a construct expressing GemC1-HA. The transfected GemC1-HA is able to co-precipitate the endogenous Geminin (Fig. 4 ▸ *c*), indicating that GemC1 and Geminin also interact in human cells. To further determine whether this interaction is dependent on the coiled-coil domain of Geminin, we made a Geminin(1–72) construct encompassing the N-terminal 72 amino acids of Geminin and lacking the coiled-coil domain, and transfected Geminin and Geminin(1–72) as GFP fusions together with GemC1-HA in U2OS cells. The transfected GemC1-HA is able to co-precipitate GFP-Geminin but not GFP-Geminin(1–72) (Fig. 4 ▸ *d*), indicating that the Geminin coiled coil is necessary for the interaction.

**Figure 5:**
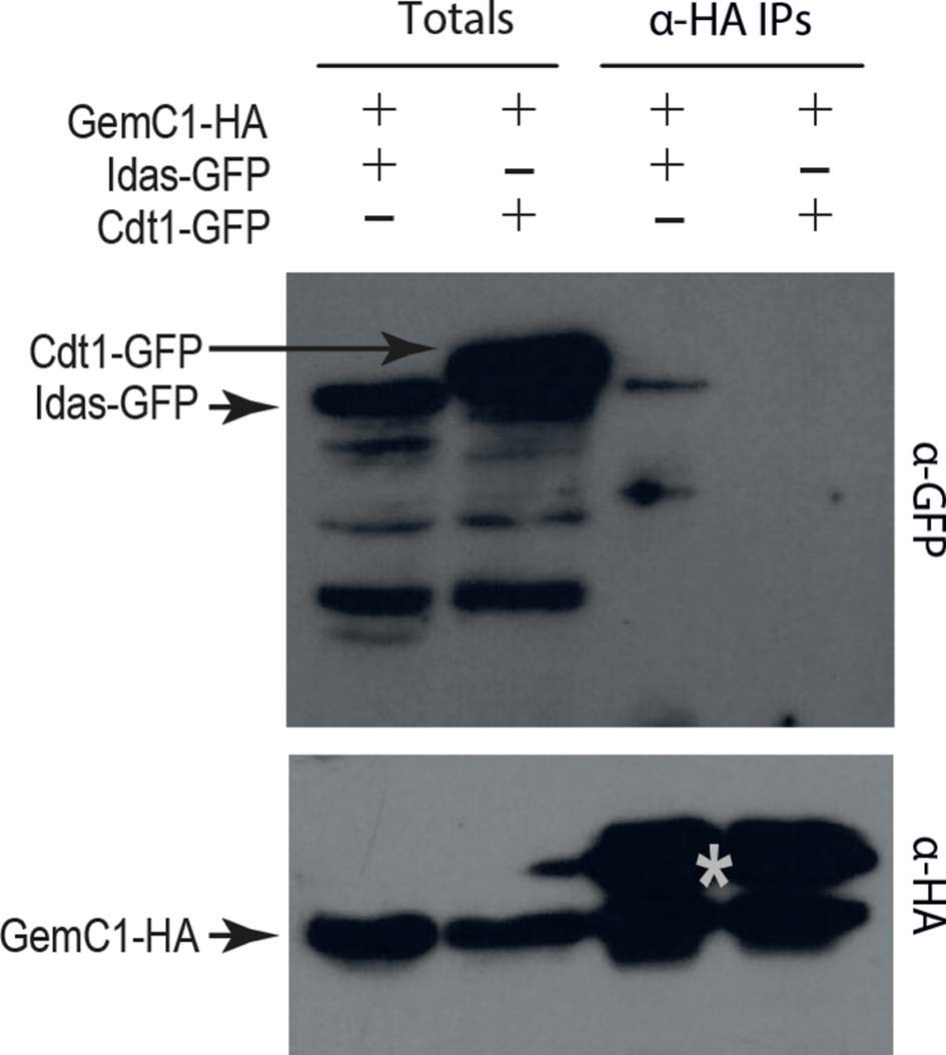
GemC1 interacts with Idas but does not interact with Cdt1. U2OS cells were co-transfected with vectors expressing GemC1-HA and Idas-GFP or Cdt1-GFP, as indicated. For each lane, following immunoprecipitation with anti-HA specific antibodies, total cell lysates and immunoprecipitates were analyzed by Western blotting with anti-GFP (upper panel) and anti-HA (lower panel) specific antibodies. In the lower panel, the grey star marks the large chain of the IgGs present in the anti-HA immunoprecipitates. The unlabelled bands in the upper panel are nonspecific bands for the weak interaction of GPA with Idas-GFP, but no interaction with Cdt1-GFP is detected.

We then wanted to check whether GemC1 also interacts with Idas. For this, we used co-transfection of U2OS cells with GemC1-HA and GFP-tagged Idas. A weak interaction between GemC1 and Idas was observed under these conditions (Fig. 5 ▸), in which GemC1-HA was only able to co-precipitate a small fraction of the total Idas-GFP protein. However, we were unable to produce any GemC1–Idas complex from bacteria for *in vitro* studies. In a parallel experiment, we also checked whether GemC1 binds Cdt1, the major partner of Geminin, but we were unable to observe an interaction.

Next, we wanted to study the stability of the GemC1 homodimers and heterodimers in comparison with other dimers formed by the Geminin-like family of proteins.

### 3.3. On the stability of the Geminin-family coiled coils   

We have collectively shown that the three family members, Geminin, Idas and GemC1, can form homodimers and that Idas and GemC1 can form heterodimers with Geminin through their coiled-coil domains. Although we were able to observe a weak Idas–GemC1 interaction in human cells, we were unable to produce any form of such a recombinant complex in order to check its stability. We have previously studied the stability of the Idas and Geminin coiled-coil dimers and concluded that the tIdas–tIdas dimer was unstable under physiological conditions, while tGeminin–tGeminin and tIdas–tGeminin were stable proteins (Caillat *et al.*, 2013 ▸).

**Figure 6:**
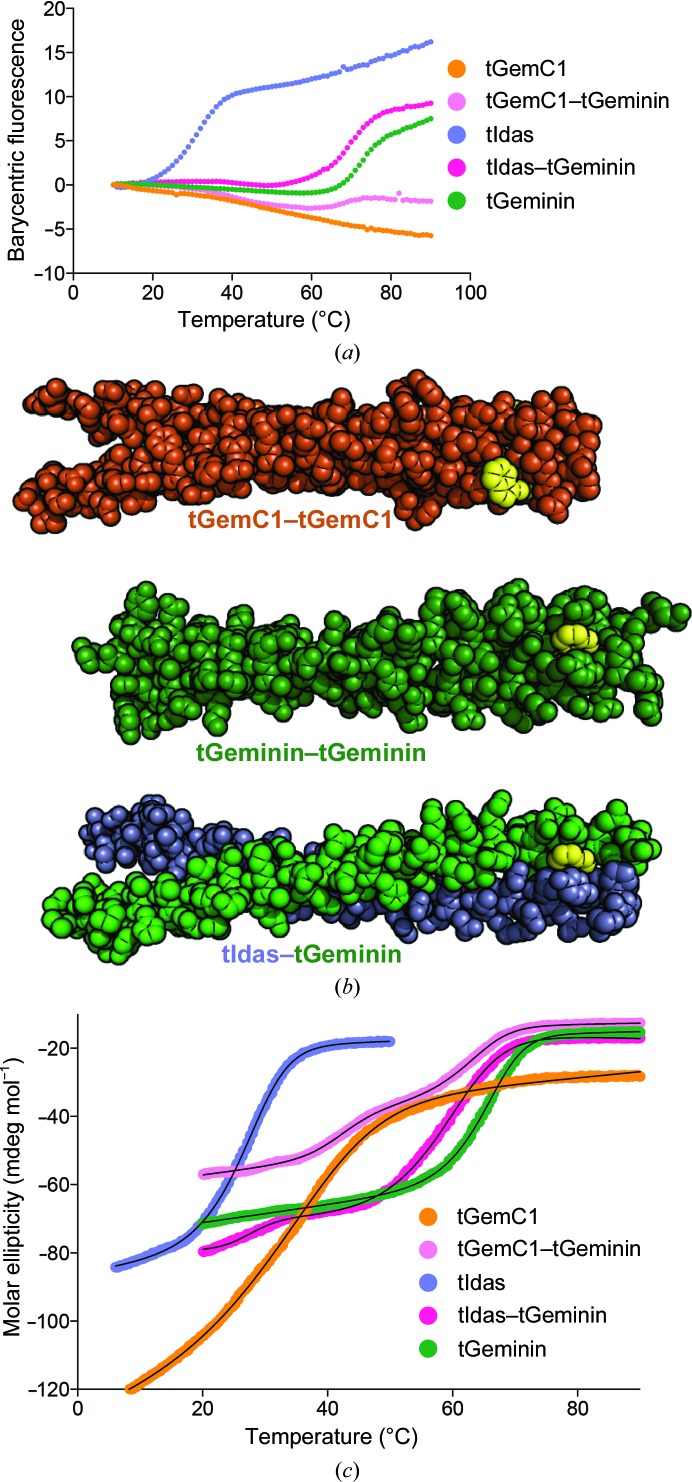
Thermal stabilities of the Geminin-family coiled coils. (*a*) Barycentric fluorescence as a function of temperature, measured in the OPTIM 1000 instrument, showing the melting point for the five available homodimers and heterodimers. (*b*) A sphere model of GemC1, Geminin and Idas–Geminin dimers, with the N-terminal tryptophan that is already solvent-accessible in GemC1 highlighted in yellow. (*c*) Molar ellipticity at 220 nm as a function of temperature as measured by circular dichroism (CD), showing the melting points for the five available homodimers and heterodimers. The thin black line represents the fitted model.

We first checked the stabilities of all five dimers (tGeminin–tGeminin, tIdas–tIdas, tGemC1–tGemC1, tIdas–tGeminin and tGemC1–Geminin) using the OPTIM 1000 instrument, monitoring tryptophan fluorescence to estimate the stability of the dimers. While we were able to accurately reproduce our previous results (Table 2 ▸), unanticipated curves were obtained for the tGemC1-containing complexes (Fig. 6 ▸ *a*). Structural information can provide a biophysical explanation for this unexpected behaviour: the hydrophobic surfaces of the Trp99 and Trp182 residues in Geminin and Idas are very well buried between neighbouring side chains of the coiled coil (Fig. 6 ▸ *b*), but in GemC1 Trp75 is surface-exposed. It is important to note that while this tryptophan is unique in the N-terminus of all three coiled-coil sequences, it is not actually conserved and is not in the same heptad nor in the same coil register (*d* in Geminin and Idas and *c* in GemC1). Thus, the environment of Trp75 in GemC1 will hardly change upon unfolding and no signal should be visible in the melting curves. Careful analysis of the melting curves supports this theory: while for the tGemC1 homodimer the signal decreases steadily without a clear deflection point, for the tGemC1–tGeminin complex there is a signal increase at about 65°C, similar to that owing to the unfolding of the tGeminin homodimer, which is likely to come from the complete melting of the Geminin chain at this temperature.

To examine the stability of GemC1 complexes without using the tryptophan-fluorescence signal, we resorted to the well established method of circular dichroism (CD). As the coiled coil is helical, we chose to study the denaturation of the helices, monitoring the ellipticity at 222 nm. It was evident from the melting curves (Fig. 6 ▸ *c*) that while the three homodimers unfold in a single state, the two heterodimers unfold in two states, with each helix presumably having a different melting point. Analyzing the data, we assumed that the coiled coils unfold as a dimer (Greenfield, 2006 ▸), as they are not interlinked by covalent bonds, in a single step or in two steps, depending on their homodimeric or heterodimeric state. This analysis clearly shows that tGemC1, with a *T*_m_ of 34.6°C, is not as stable as tGeminin (65.3°C) but is significantly more stable than Idas (26.9°C). Still, this value suggests that the tGemC1 homodimer should be rather unstable in physiological environments; as we have not been able to obtain soluble full-length tGemC1 to perform this experiment, we cannot be confident whether this conclusion can be extended to the wild-type protein. However, our data suggest that GemC1 alone may be unstable and may be unlikely to be present in cells as a homodimer on its own under physiological conditions. We speculate that GemC1 may exist as a heterodimer with Geminin in cells, while complex formation with other partners, or post-translational modification, may be required to stabilize a GemC1 homodimer. The tGemC1–tGeminin complex shows a two-state unfolding: the first event is at 42.6°C and the second event at 62.6°C. As we obtain an excellent fit presuming that that the ratio between the two events is equal to the ratio of total change in the ellipticity of unfolding between tGemC1 and tGeminin, we interpret the first event as the unfolding of tGemC1 (which has been stabilized by about 8°C owing to interaction with Geminin) and the second event as the unfolding of tGeminin, which has been moderately destabilized. This result implies that when GemC1 and Geminin are co-expressed in cells the predominant form of GemC1 is likely to be in complex with Geminin, as previously suggested for Idas. Interestingly, the CD data also show a two-event curve for the tIdas–tGeminin complex. However, in this case the associated molar ellipticity change for the first event is very small and we think that this is likely to be an unfolding of the tIdas C-terminus that is not part of the coiled coil; the two helices in the tIdas–tGeminin complex unfold at similar temperatures, but the data cannot be deconvoluted. The same could hold true for the tGemC1–tGeminin unfolding but to a much lesser degree, as there we more clearly see the two states which are more likely to correspond to the two helices.

Some interesting hypotheses could be extracted by comparing the OPTIM and CD experiments: owing to the positioning of the tryptophan residue OPTIM monitors the unfolding of the N-terminal region of the coiled coils, while the CD gives a more global picture. For tGeminin and tIdas, it is clear that the *T*_m_ obtained from OPTIM (corresponding to the N-terminal part) is higher than that obtained from CD (corresponding to the complete coiled coil): this could imply that these coiled coils unfold from the C-terminus towards the N-terminus. Presuming that the folding takes the same pathway as the unfolding that we study here, this would in turn imply that these coils also fold from the N-terminus towards the C-terminus, favouring our previous hypothesis of co-translational assembly of the heterodimeric Idas–Geminin complex (Caillat *et al.*, 2013 ▸) and leading us to propose that the same holds true for the GemC1–Geminin complex. The biophysical issues around coiled-coil folding are considerable (for a review, see Lupas & Gruber, 2005 ▸) and sophisticated approaches have been used to study these problems. Thus, the above conjecture should be taken with caution. However, we believe that monitoring the unfolding through the two different signals that we use in this case (for the first time, to our knowledge) provides novel insight into how the Geminin-family coiled coils might fold.

In conclusion, our structure of the GemC1 coiled coil, together with biophysical data, suggest that the GemC1 coiled coil is likely to be unstable and (as for Idas) a GemC1–Geminin dimer might be a more stable structure in cells. Our results thus reinforce the concept that both Idas and GemC1 may modulate the abundance of the Geminin dimer when co-expressed in cells. The GemC1–Geminin and Idas–Geminin heterodimers are likely to be major pools of Idas and GemC1 in cells which co-express Geminin, such as proliferating cells, and could modulate the diverse functions of Geminin, Idas and GemC1 in proliferation and differentiation.

## Supplementary Material

PDB reference: GEMC1 coiled-coil domain, 5c9n

## Figures and Tables

**Table 1:** Crystallographic data Values in parentheses are for the highest resolution bin.

Data collection	
Space group	*P*2_1_2_1_2_1_
Unit-cell parameters (, )	*a* = 50.03, *b* = 70.57, *c* = 83.06
Resolution ()	53.82.20 (2.322.20)
*R*_merge_	0.070 (0.871)
*I*/(*I*)	11.8 (1.5)
Completeness (%)	99.5 (99.8)
Multiplicity	3.7 (3.8)
Refinement	
Resolution ()	53.82.20
No. of reflections	15376
*R*_work_/*R*_free_ (%)	22.3/24.9
No. of atoms	
Protein	1146
Ligand/ion	16/0
Water	37
*B* factors (^2^)	
Wilson	45.03
Average of atoms	36.89
R.m.s. deviations	
Bond r.m.s.d. ()/r.m.s.*Z*	0.010/0.502
Angle r.m.s.d. ()/r.m.s.*Z*	1.246/0.586
Validation (*MolProbity*)	
Ramachandran favoured (%)	100
Ramachandran outliers (%)	0
*MolProbity* score	1.72 [100th percentile]

**Table 2:** Thermostability data from tryptophan-fluorescence (OPTIM) and circular-dichroism (CD) experiments n.d., not determined; n.a., not applicable.

	*T*_m_(OPTIM) (C)	*T*_m_(CD) (C)	*T*_m2_(CD) (C)	*H*_1_ (kcalmol^1^)	*H*_2_ (kcalmol^1^)
tGemC1	n.d.	34.6 0.2	n.a.	35.6 0.4	n.a.
tGemC1tGeminin	n.d.	42.5 0.1	63.2 0.1	31.4 0.2	116.0 1.9
tIdas	30.4 0.3	26.9 0.03	n.a.	72.4 0.5	n.a.
tIdastGeminin	69.4 0.3	(27.6 0.1)	58.7 0.1	n.d.	n.d.
tGeminin	71.2 0.1	65.3 0.02	n.a	101.8 0.4	n.a.

## References

[bb1] Afonine, P. V., Grosse-Kunstleve, R. W., Echols, N., Headd, J. J., Moriarty, N. W., Mustyakimov, M., Terwilliger, T. C., Urzhumtsev, A., Zwart, P. H. & Adams, P. D. (2012). *Acta Cryst.* D**68**, 352–367. 10.1107/S0907444912001308 PMC3322595 22505256

[bb2] Balestrini, A. A., Cosentino, C. C., Errico, A. A., Garner, E. E. & Costanzo, V. V. (2010). *Nature Cell Biol.* **12**, 484–491. 10.1038/ncb2050 PMC2875115 20383140

[bb3] Caillat, C., Pefani, D.-E., Gillespie, P. J., Taraviras, S., Blow, J. J., Lygerou, Z. & Perrakis, A. (2013). *J. Biol. Chem.* **288**, 31624–31634. 10.1074/jbc.M113.491928 PMC3814758 24064211

[bb4] Caillat, C. & Perrakis, A. (2012). *Subcell. Biochem.* **62**, 71–87. 10.1007/978-94-007-4572-8_5 22918581

[bb5] Champeris Tsaniras, S., Kanellakis, N., Symeonidou, I. E., Nikolopoulou, P., Lygerou, Z. & Taraviras, S. (2014). *Semin. Cell Dev. Biol.* **30**, 174–180. 10.1016/j.semcdb.2014.03.013 24641889

[bb6] De Marco, V. *et al.* (2009). *Proc. Natl Acad. Sci. USA*, **106**, 19807–19812.

[bb7] Emsley, P., Lohkamp, B., Scott, W. G. & Cowtan, K. (2010). *Acta Cryst.* D**66**, 486–501. 10.1107/S0907444910007493 PMC2852313 20383002

[bb8] Greenfield, N. J. (2006). *Nature Protoc.* **1**, 2527–2535. 10.1038/nprot.2006.204 PMC2752288 17406506

[bb9] Grigoryan, G. & Degrado, W. F. (2011). *J. Mol. Biol.* **405**, 1079–1100. 10.1016/j.jmb.2010.08.058 PMC3052747 20932976

[bb32] Iliou, M. S., Kotantaki, P., Karamitros, D., Spella, M., Taraviras, S. & Lygerou, Z. (2013). *Mech. Ageing Dev.* **134**, 10–23. 10.1016/j.mad.2012.10.001 23142824

[bb10] Joosten, R. P., Long, F., Murshudov, G. N. & Perrakis, A. (2014). *IUCrJ*, **1**, 213–220. 10.1107/S2052252514009324 PMC4107921 25075342

[bb11] Kabsch, W. (2010). *Acta Cryst.* D**66**, 125–132. 10.1107/S0907444909047337 PMC2815665 20124692

[bb12] Langer, G., Cohen, S. X., Lamzin, V. S. & Perrakis, A. (2008). *Nature Protoc.* **3**, 1171–1179. 10.1038/nprot.2008.91 PMC2582149 18600222

[bb13] Lee, C., Hong, B., Choi, J., Kim, Y., Watanabe, S., Ishimi, Y., Enomoto, T., Tada, S., Kim, Y. & Cho, Y. (2004). *Nature (London)*, **430**, 913–917. 10.1038/nature02813 15286659

[bb14] Luna-Vargas, M. P. A., Christodoulou, E., Alfieri, A., van Dijk, W. J., Stadnik, M., Hibbert, R. G., Sahtoe, D. D., Clerici, M., Marco, V. D., Littler, D., Celie, P. H. n., Sixma, T. K. & Perrakis, A. (2011). *J. Struct. Biol.* **175**, 113–119. 10.1016/j.jsb.2011.03.017 21453775

[bb15] Lupas, A. N. & Gruber, M. (2005). *Adv. Protein Chem.* **70**, 37–78. 10.1016/S0065-3233(05)70003-6 15837513

[bb16] Lygerou, Z. & Nurse, P. (2000). *Science*, **290**, 2271–2273. 10.1126/science.290.5500.2271 11188727

[bb17] Ma, L., Quigley, I., Omran, H. & Kintner, C. (2014). *Genes Dev.* **28**, 1461–1471. 10.1101/gad.243832.114 PMC4083089 24934224

[bb18] McCoy, A. J. (2007). *Acta Cryst.* D**63**, 32–41. 10.1107/S0907444906045975 PMC2483468 17164524

[bb19] McGarry, T. J. & Kirschner, M. W. (1998). *Cell*, **93**, 1043–1053. 10.1016/s0092-8674(00)81209-x 9635433

[bb20] Murshudov, G. N., Skubák, P., Lebedev, A. A., Pannu, N. S., Steiner, R. A., Nicholls, R. A., Winn, M. D., Long, F. & Vagin, A. A. (2011). *Acta Cryst.* D**67**, 355–367. 10.1107/S0907444911001314 PMC3069751 21460454

[bb21] Newman, J., Egan, D., Walter, T. S., Meged, R., Berry, I., Ben Jelloul, M., Sussman, J. L., Stuart, D. I. & Perrakis, A. (2005). *Acta Cryst.* D**61**, 1426–1431. 10.1107/S0907444905024984 16204897

[bb22] Pefani, D.-E., Dimaki, M., Spella, M., Karantzelis, N., Mitsiki, E., Kyrousi, C., Symeonidou, I. E., Perrakis, A., Taraviras, S. & Lygerou, Z. (2011). *J. Biol. Chem.* **286**, 23234–23246. 10.1074/jbc.M110.207688 PMC3123090 21543332

[bb23] Saxena, S., Yuan, P., Dhar, S. K., Senga, T., Takeda, D., Robinson, H., Kornbluth, S., Swaminathan, K. & Dutta, A. (2004). *Mol. Cell*, **15**, 245–258. 10.1016/j.molcel.2004.06.045 15260975

[bb24] Seo, S. & Kroll, K. L. (2006). *Cell Cycle*, **5**, 374–379. 10.4161/cc.5.4.2438 16479171

[bb25] Stubbs, J. L., Vladar, E. K., Axelrod, J. D. & Kintner, C. (2012). *Nature Cell Biol.* **14**, 140–147. 10.1038/ncb2406 PMC3329891 22231168

[bb30] Symeonidou, I. E., Kotsantis, P., Roukos, V., Rapsomaniki, M. A., Grecco, H. E., Bastiaens, P., Taraviras, S. & Lygerou, Z. (2013). *J. Biol. Chem.* **288**, 35852–35867. 10.1074/jbc.M113.474825 PMC3861635 24158436

[bb26] Tada, S., Li, A., Maiorano, D., Méchali, M. & Blow, J. J. (2001). *Nature Cell Biol.* **3**, 107–113. 10.1038/35055000 PMC3605706 11175741

[bb27] Thépaut, M., Maiorano, D., Guichou, J.-F., Augé, M.-T., Dumas, C., Méchali, M. & Padilla, A. (2004). *J. Mol. Biol.* **342**, 275–287. 10.1016/j.jmb.2004.06.065 15313623

[bb28] Walshaw, J. & Woolfson, D. N. (2001). *J. Mol. Biol.* **307**, 1427–1450. 10.1006/jmbi.2001.4545 11292353

[bb29] Wohlschlegel, J. A., Dwyer, B. T., Dhar, S. K., Cvetic, C., Walter, J. C. & Dutta, A. (2000). *Science*, **290**, 2309–2312. 10.1126/science.290.5500.2309 11125146

[bb31] Xouri, G., Lygerou, Z., Nishitani, H., Pachnis, V., Nurse, P. & Taraviras, S. (2004). *Eur. J. Biochem.* **271**, 3368–3378. 10.1111/j.1432-1033.2004.04271.x 15291814

